# Simultaneous prosthodontic treatment for crowns of natural teeth and dental implants using digital technology: A case report

**DOI:** 10.1002/ccr3.7187

**Published:** 2023-05-16

**Authors:** Akinori Tasaka, Hodaka Sasaki, Atsuro Harada, Kosei Ito, Hiro Kobayashi, Takahiro Shimizu, Shuichiro Yamashita

**Affiliations:** ^1^ Department of Removable Partial Prosthodontics Tokyo Dental College Tokyo Japan; ^2^ Department of Oral and Maxillofacial Implantology Tokyo Dental College Tokyo Japan

**Keywords:** CAD/CAM, crown prosthesis, digital technology, implant treatment, intraoral scanner

## Abstract

The aim of this clinical report was to describe the improvement of masticatory disorders with the use of digital technology to simultaneously perform prosthodontic treatment of natural teeth and edentulous areas. Computer‐guided implant surgery was performed, and crown prostheses and implant superstructures were fabricated simultaneously using digital technology.

## 1 INTRODUCTION

Fabricating crowns from imaging data obtained by an intra‐oral scanner (IOS) is becoming more common.^1^, ^2^, ^3^ It has been reported that crowns and bridges fabricated using an IOS show no difference in fit compared to conventional methods using impression materials.^4^ In implant treatment, the combination of intraoral scanning data and CT data enables planning of implant placement, the fabrication of surgical guide templates, and provisional restorations in advance. In addition, a definitive prosthesis can be fabricated from intraoral scanning data with the implant scan body connected on the implant body after implant placement.^5^, ^6^ Thus, implant treatment can be completely performed using a digital process from diagnosis to final prosthodontic treatment.

There have been many reports of cases in which the crown of a natural tooth or implant treatment was performed independently using digital technology. However, there are few case reports of the use of digital technology in prosthodontic treatment planning including both simultaneously. The combination of natural teeth and implant complicates the prosthodontic treatment, multi abutment impression can be difficult^7^ and repeated removal and replacement of healing abutments result in frequent injuries to the soft tissues.^8^ In this paper, implant bodies were placed by guided surgery, intraoral scanning was performed, “cut out‐rescan” technique was used for the natural teeth and scan body was used for the implant on never removed abutment, and definitive prosthesis made of zirconia were fabricated from the data obtained. The aim of this clinical report was to describe the improvement of masticatory disorders by using digital technology to simultaneously perform prosthodontic treatment of natural teeth and of edentulous areas.

## 2 CASE DESCRIPTION AND RESULTS

**FIGURE 1:**
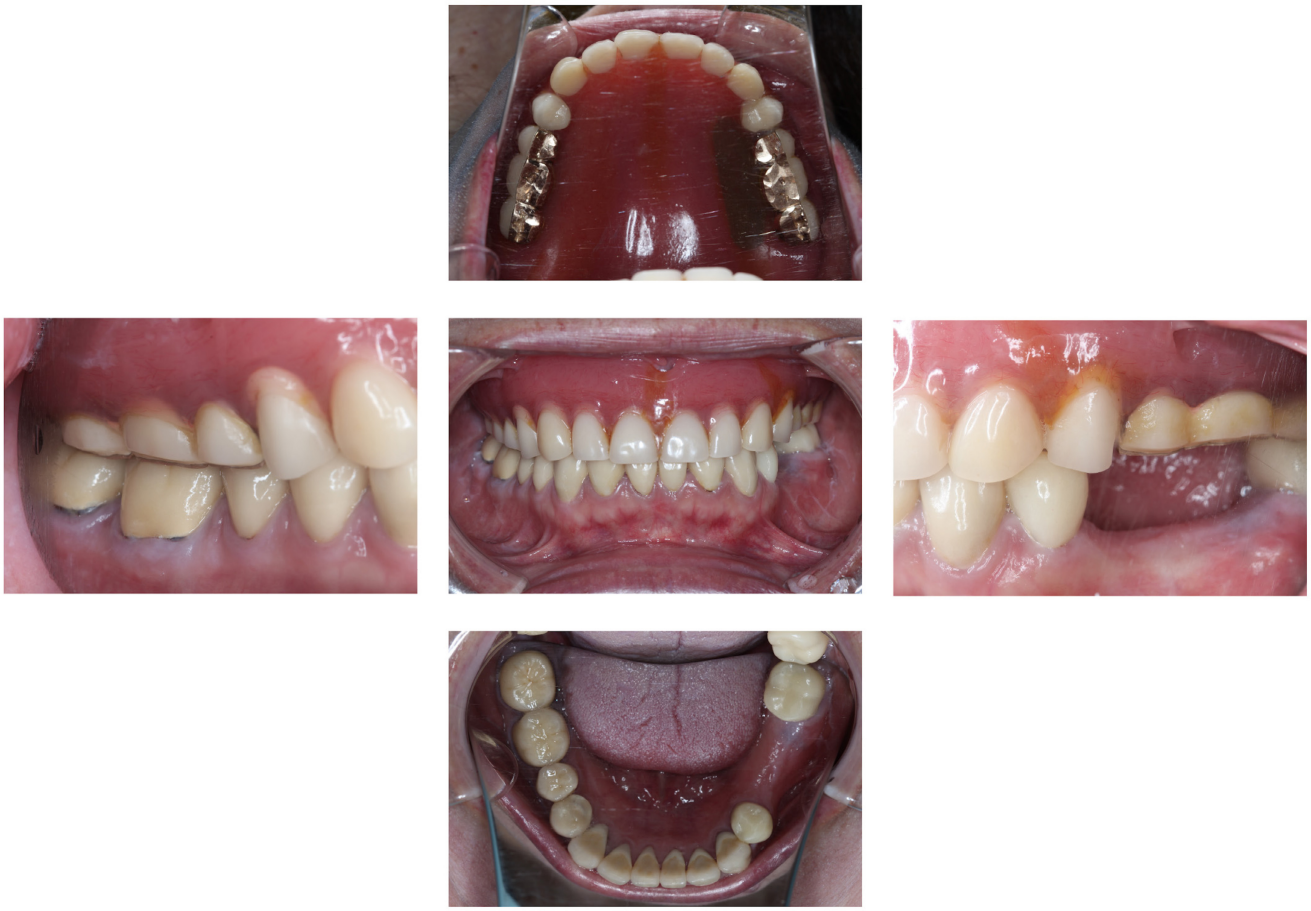
Intraoral view before prosthodontic treatment.

**FIGURE 2:**
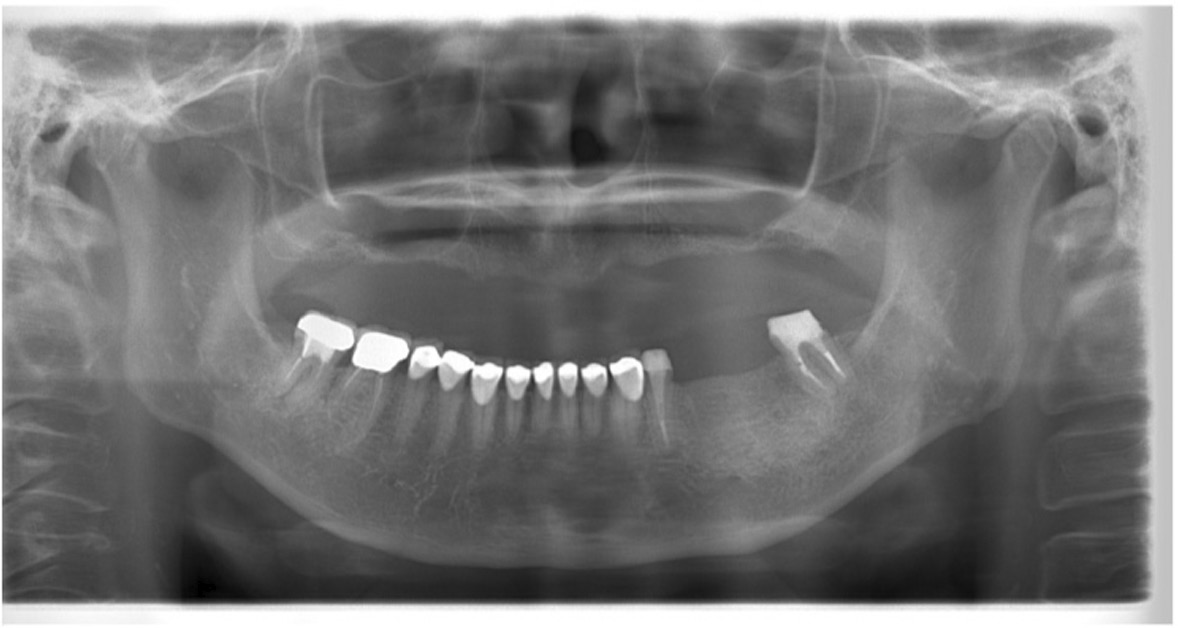
Panoramic radiography obtained before treatment.

A 56‐year‐old woman visited the Department of Prosthodontics of Suidobashi Hospital at Tokyo Dental College complaining of masticatory difficulty due to missing mandibular left second premolar and first molar teeth (#35 and #36: FDI two‐digit system). Wear on the metal tooth and fracture of the porcelain tooth of the maxillary complete denture were observed, which were repaired in the midline of the denture base. A temporary crown was placed on #35 and #36. All mandibular teeth were restored with crowns, and the veneer of #44 was fractured. There was no recorded mobility for the remaining teeth, with the periodontal pocket found to be less than 3 mm (Figures 1 and 2). An evaluation of chewing function^9^ was level 3, with a masticatory score of 42%. The result of the test for chewing ability using gummy jellies^10^ was 133 mg/dL.

**FIGURE 3:**
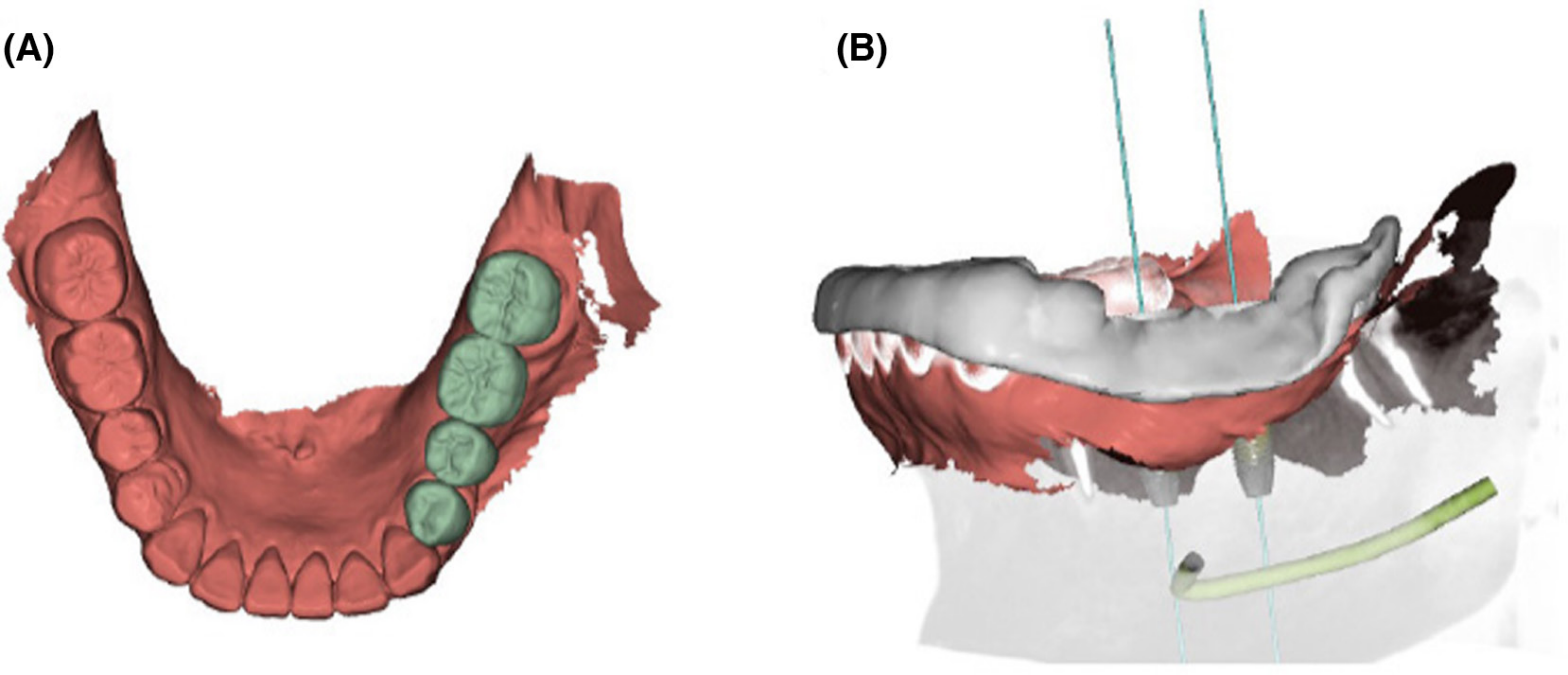
Prosthodontic treatment planning. (A) Virtual wax‐up. (B) Surgical guide design.

**FIGURE 4:**
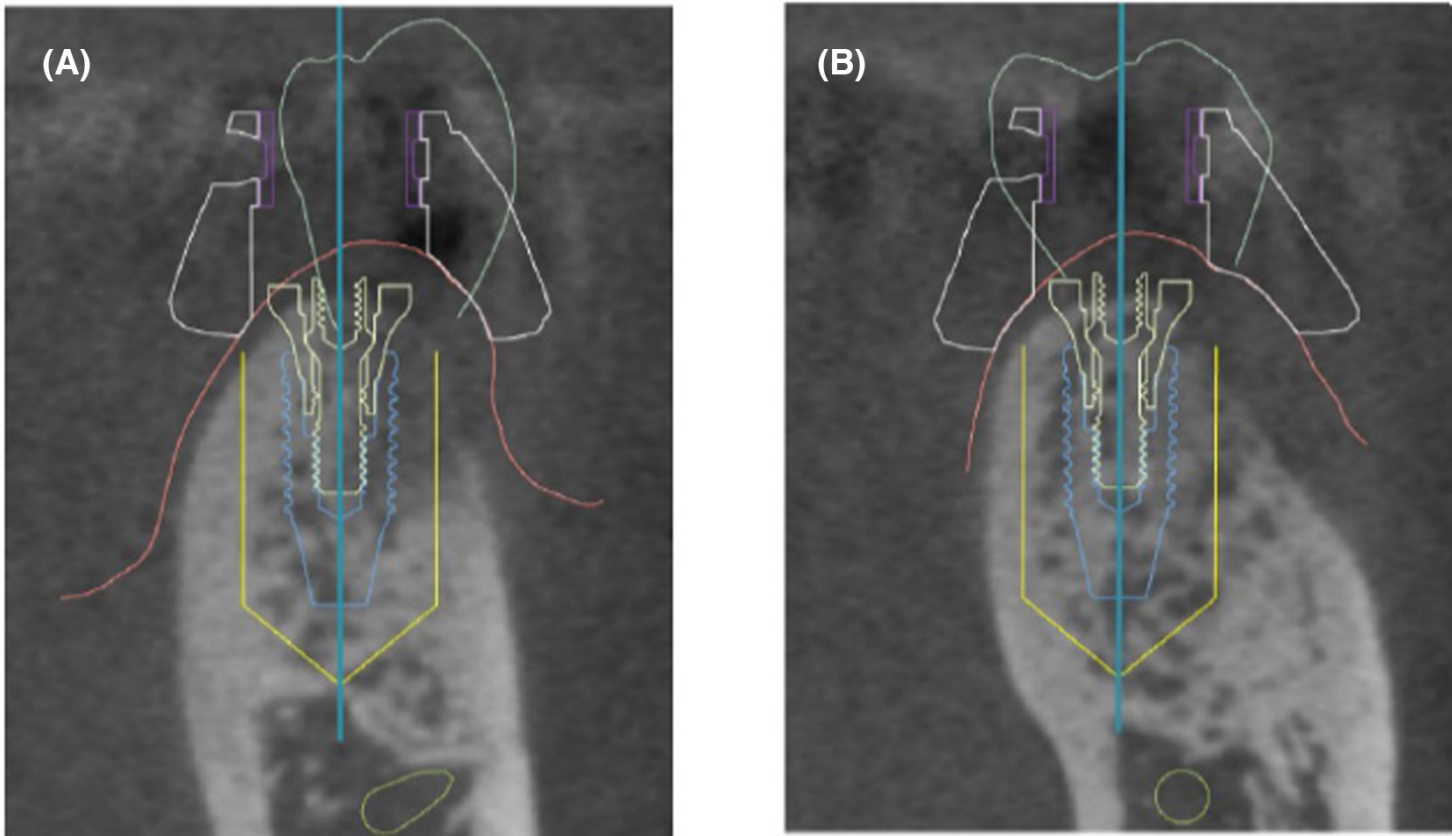
Implant placement planning. (A) Coronal view of the designed implant position for the second premolar. (B) Coronal view of the designed implant position for the first molar.

The present case was classified as level III using the classification system for prosthodontic treatment by the Japanese Society of Prosthodontics. The anatomical structure measured by CT scan showed buccolingual width greater than 6 mm, and the distance from the alveolar crest to the mandibular canal was greater than 13 mm at both the #35 and #36 areas. The implant placement was planned using dental implant planning software (DTX Studio, Nobel Biocare) with the intraoral scanning data and CT data, and two implant bodies were planned to be placed by guided surgery (Figures 3 and 4). The treatment plan for the final superstructure, including #34 and #37 crown prostheses, was explained to the patient, and consent was obtained regarding the fabrication of a zirconia crown based on intraoral scanning data. The patient did not wish to have the #44 crown refabricated.

**FIGURE 5:**
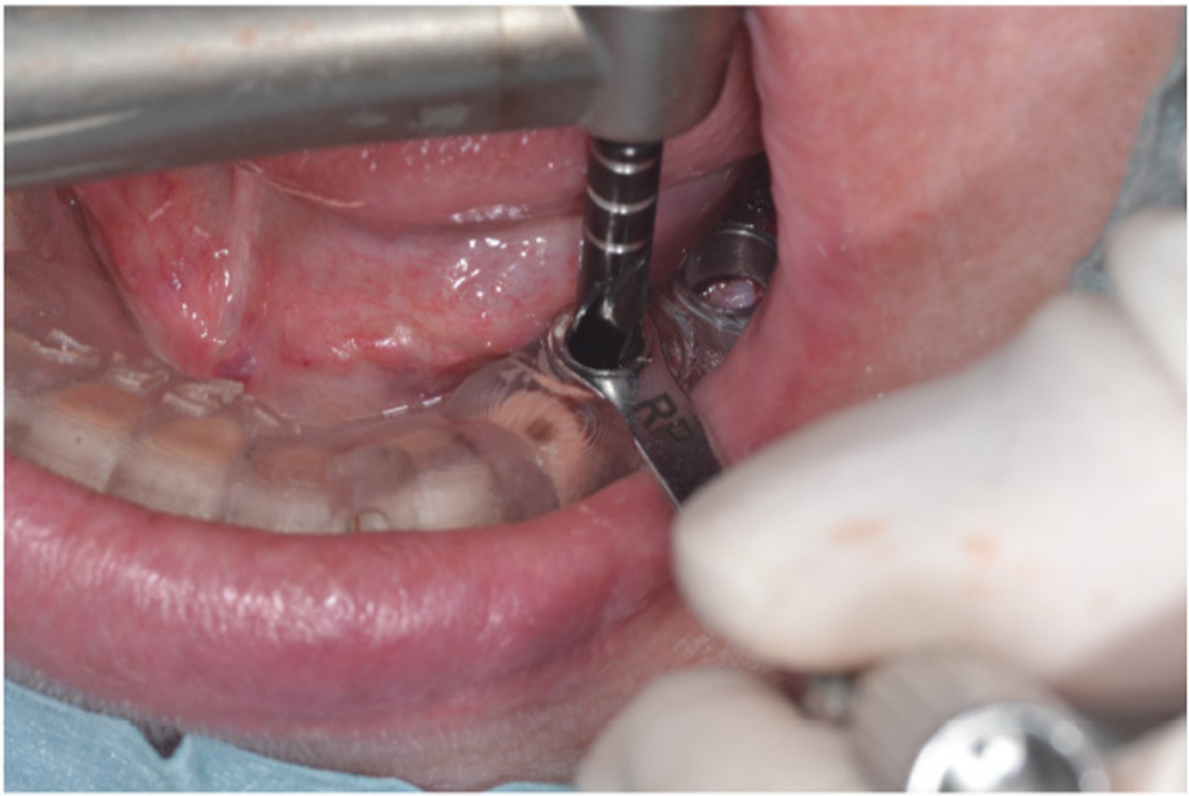
Intraoperative photograph of implant placement.

The implant socket was formed using a surgical guide at #35 and #36, and two implant bodies (NobelParallel CC RP φ4.3 × 10 mm Nobel Biocare) were placed. A one‐stage implant placement procedure was selected, and the abutment (On1 base abutment, Nobel Biocare) was fastened with a torque of 35 N/cm (Figure 5).

**FIGURE 6:**
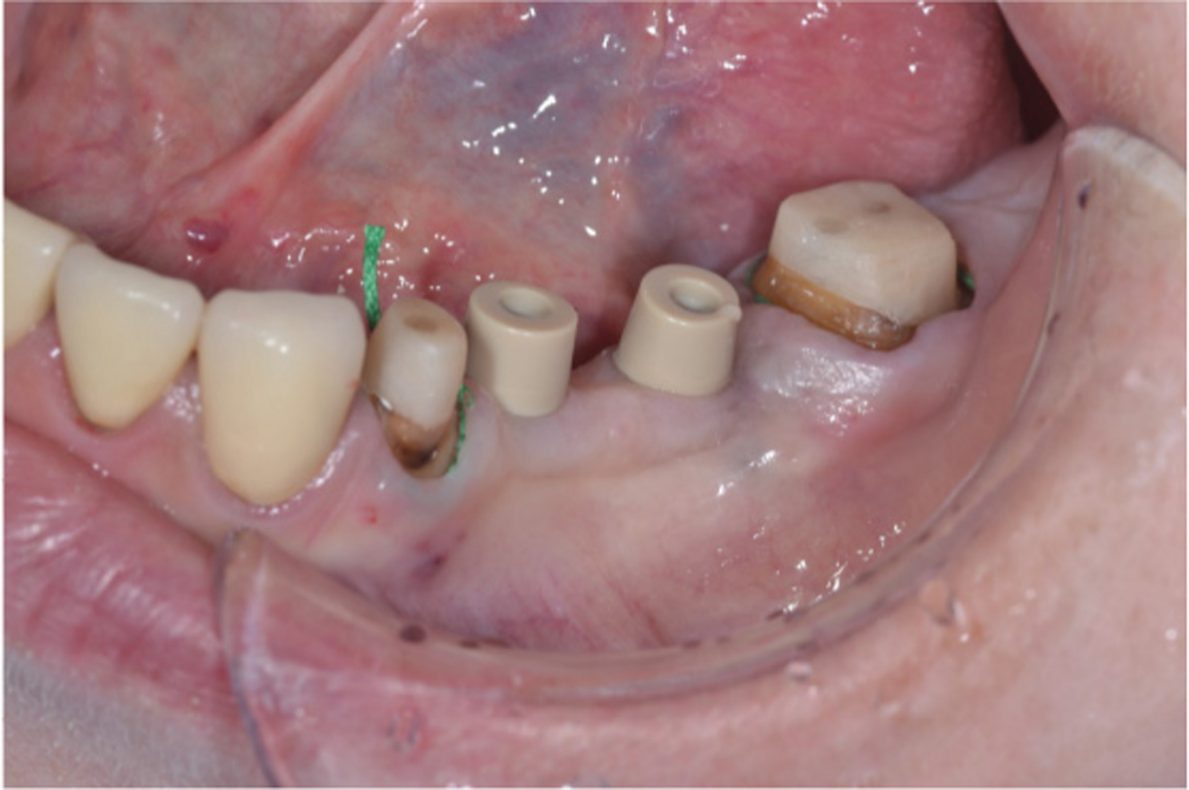
Abutment teeth after preparation and attached scan body to implant abutments.

**FIGURE 7:**
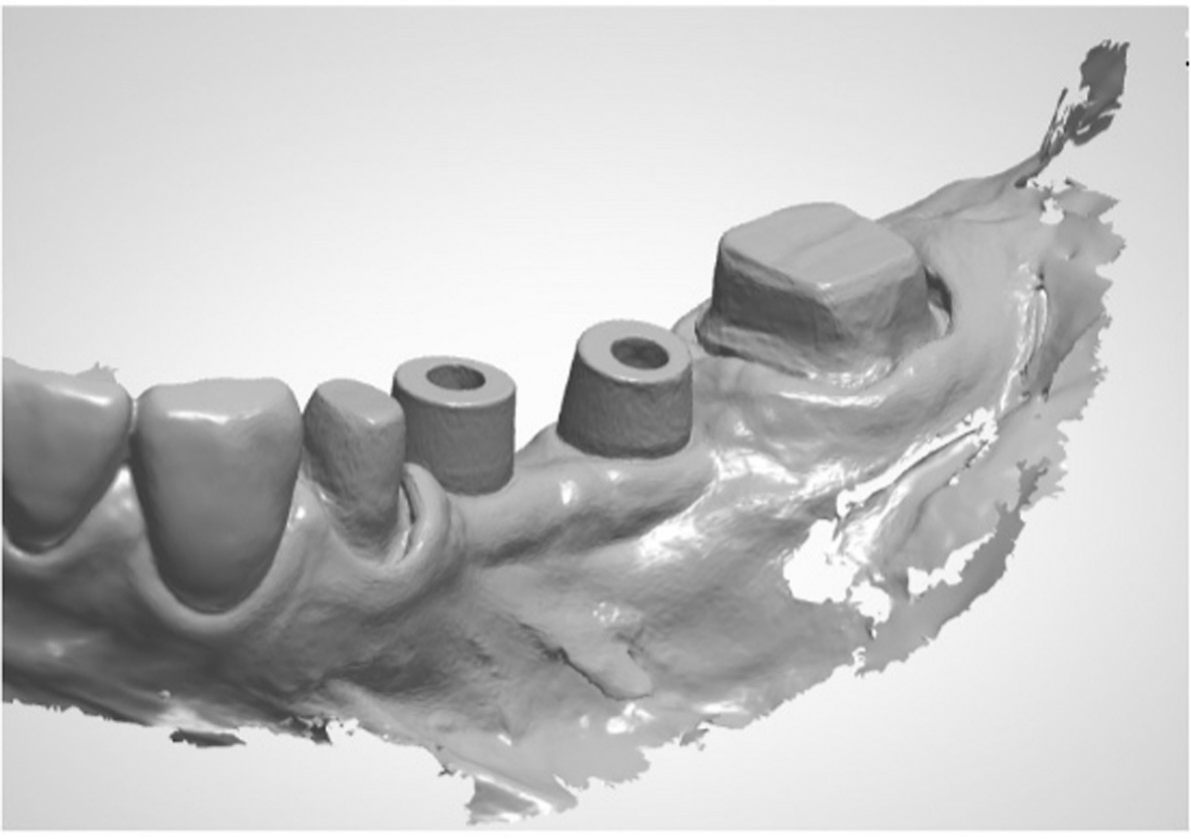
Intraoral scanning data.

Three months after implant placement, the peri‐implant tissues were confirmed to be in good condition, and abutment tooth preparation was performed on #34 and #37 with a minimum 1‐mm occlusal reduction and convergence angle of 6 degrees, with a deep chamfer finish line 0.5 mm below the gingival margin. Next, intraoral scanning was performed using the IOS to fabricate the provisional restorations for the abutment teeth at #34 and #37 and the implants at #35 and #36. Based on the intraoral scanning data obtained, the provisional restoration was milled using a 5‐axis milling machine (DWX‐51DC, Roland) with a resin disk (Temp, Aidite). A 3D‐printed dental model (P pro Master Model Dark beige, Straumann) was fabricated using a 3D printer (CARES P series P30, Straumann) and temporary cylinders (On1 Temporary cylinders, Nobel Biocare), and provisional restorations were fitted on the model with autopolymerizing acrylic resin. After wearing the provisional restoration for 3 months and confirming that there were no problems with occlusion, esthetics, or cleaning, fabrication of the definitive prostheses was started. Gingival retraction of the abutment teeth was carried out using the double‐cord technique, and the scan body (On1 IOS cap, Nobel Biocare) was attached to the abutment of the implants (Figure 6). Intraoral scanning was first performed with full arch scanning. Next, the imaging data of the abutment teeth and surrounding gingiva were deleted using a cutting tool. The scanning data were locked with the lock tool to avoid overwriting the captured geometry for the image data other than the abutment teeth. The secondary retraction cord of abutment teeth was removed, and the area was immediately rescanned to capture more accurate imaging data of the finish lines of the abutment teeth (Figure 7). This method of intraoral scanning is called “cut out ‐rescan” technique. The maxillary denture was scanned extraorally as the data of the antagonist arch. An IOS was used to obtain digital scans of the occlusion with the denture and provisional restoration placement. The verification jig was created to confirm the positional relationship between implants.

**FIGURE 8:**
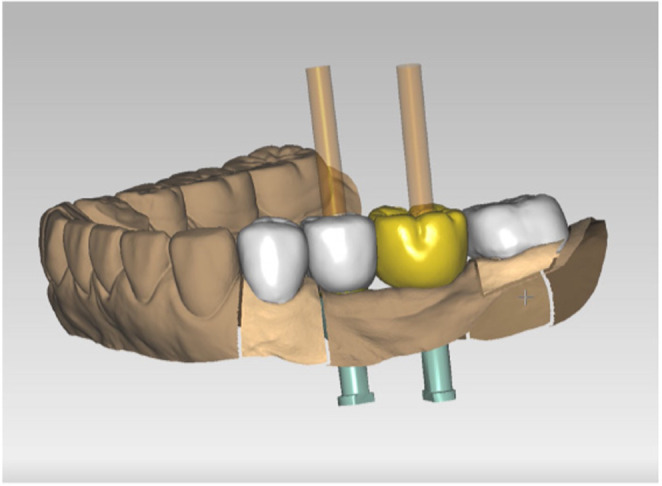
Design of final prosthesis.

**FIGURE 9:**
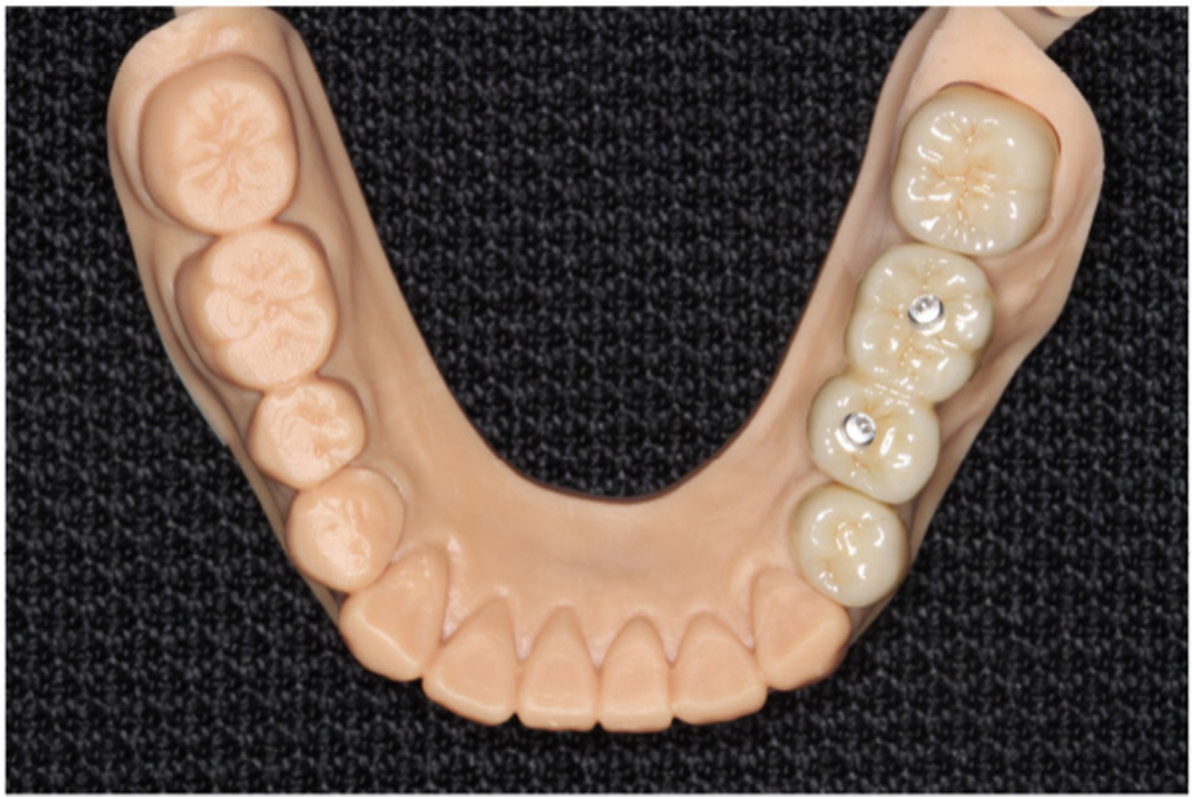
Zirconia crowns and final superstructures.

**FIGURE 10:**
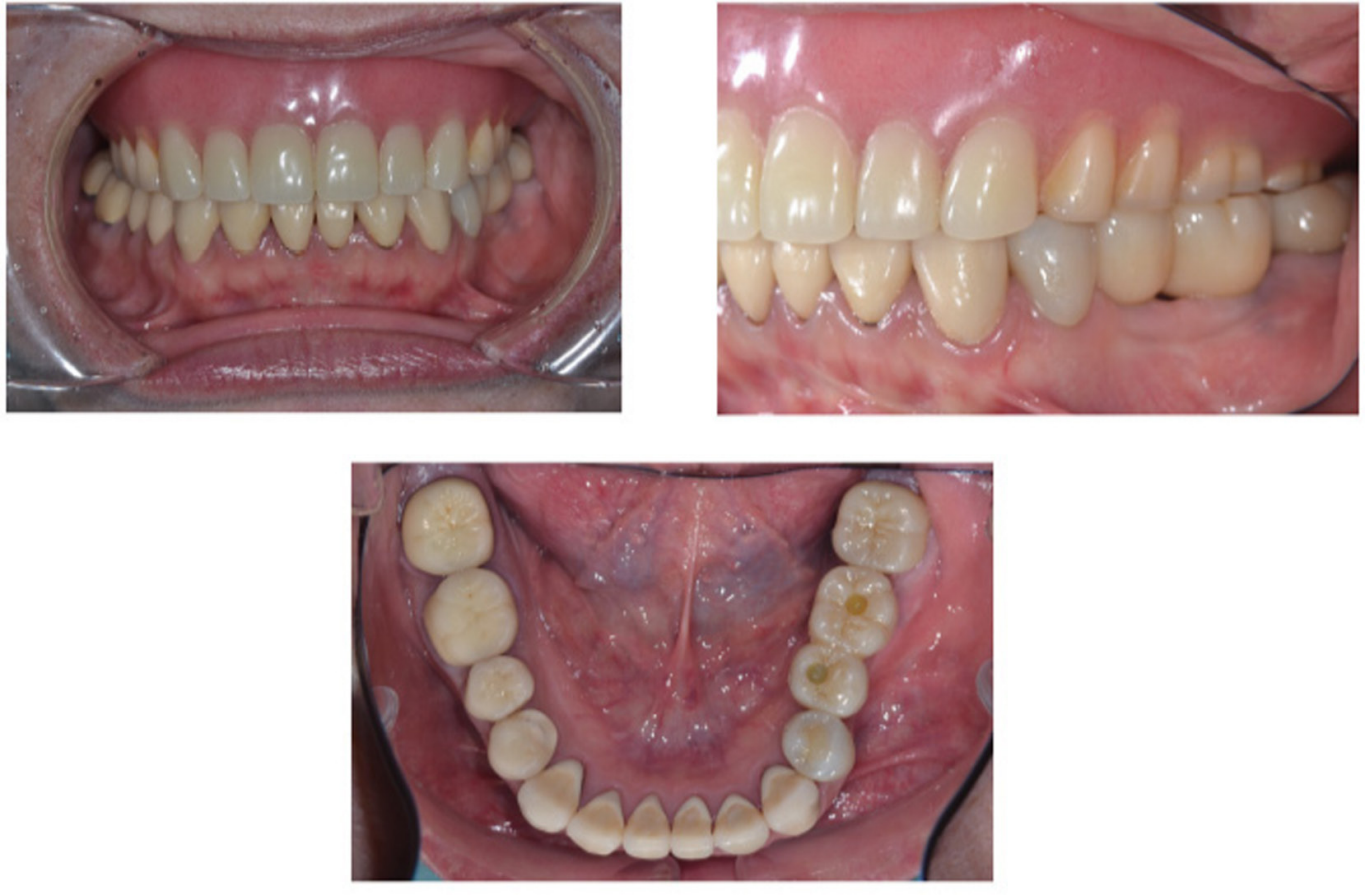
Intraoral view after prosthodontic treatment.

**FIGURE 11:**
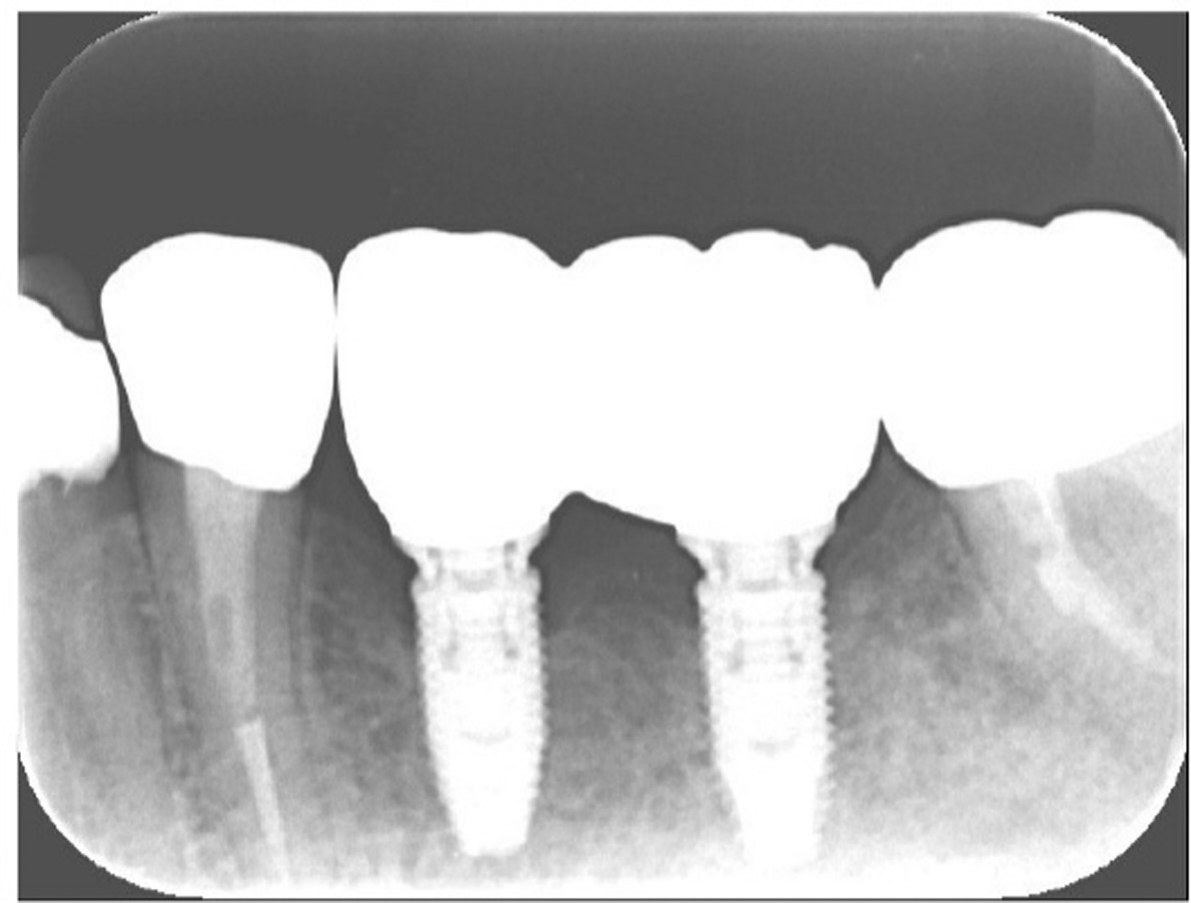
X‐ray images after treatment.

Based on the data, the crowns and implant superstructures were milled from a zirconia disk (Sakura zirconia disk, Straumann) using the 5‐axis milling machine. The crowns were designed with monolithic zirconia and the superstructures with a connected, screw‐retained–type crown (Figure 8). For the final superstructure, a zirconia crown was bonded to a titanium cylinder (On1 Universal Cylinder, Nobel Biocare) and completed (Figure 9). The superstructures were fastened with a torque of 35 N/cm, and the crowns were subsequently cemented with adhesive resin cement (RelyX Adhesive Resin Cement, 3M ESPE). After the mandibular treatment, the patient requested that the maxillary complete denture be refabricated, so the denture was fabricated using the conventional method, and the treatment was completed (Figures 10 and 11).

At the end of treatment, the masticatory score increased to 68.4%, level 4. The results of the chewing ability test with gummy jelly were 219 mg/dL, and impaired masticatory function was improved. There were no complaints of discomfort with the prosthesis. At the last maintenance 1 year after treatment, plaque control and denture cleaning were good, and there were no problems with the periodontal tissues of the remaining teeth or the peri‐implant tissues.

## 3 DISCUSSION

The development of implant simulation software has enabled surgeons to plan the ideal number, type, length, diameter, placement location, and direction of implants, assuming the location of the superstructure. In addition, the use of a surgical guide template fabricated according to the plan makes it possible to place implants more accurately.^11^ However, it should be noted that guided surgery may cause errors between the simulated position and the position of the implant after placement. The tooth‐supported surgical guide used in the present case had the highest accuracy, followed by the type using a fixed pin for mucosa support, then the mucosa‐supported type, and the bone‐supported type had the lowest accuracy, and the effect of the fixation method of the guide has been reported.^12^ Flapless guided surgery can also shorten operative time and reduce surgical invasiveness. However, in the present case, since the placement position needed to be deeper than the alveolar crest to secure the bone around the implant body, a flap was formed to remove excess bone around the implant after placement to ensure the abutment fastening. Intermittent drilling was kept in mind because of the risk of bone burn due to inadequate, deficient irrigation when using a surgical guide to create the implant socket. In some cases, there is concern about the handpiece head contacting the antagonist teeth, but this was not a problem in the present case because the maxilla was edentulous.

In the present case, digital technology was used from impression‐taking to fabrication of the definitive prosthesis. The use of an IOS for impression‐taking enabled efficient impression‐taking of implants and natural teeth. With the conventional method using silicone impression materials, the success or failure of the impression‐taking could not be evaluated until the impression was removed. However, in the present case, after full arch scanning including the abutment teeth and scan bodies using an IOS, the imaging data of the abutment teeth could be accurately obtained by applying “cut out‐rescan.” Reich et al. reported that “cut out‐rescan” technique had no effect on full arch scanning, and the IOS provides options that cannot be applied when using conventional impressions.^13^ In addition, the conventional method using bite registration materials tends to cause jaw deviation because of the loss of posterior occlusal support area. By using an IOS, interocclusal registration could be performed with provisional restoration placement, thus achieving accurate recording of occlusal relationships. The verification jig was also created to confirm the positional relationship between the implant analogs on the 3D model and the implants in the oral cavity. During fabrication of the definitive prosthesis, the data of the provisional restoration were superimposed on the CAD software to give a crown morphology that reflected the provisional restoration.

**FIGURE 12:**
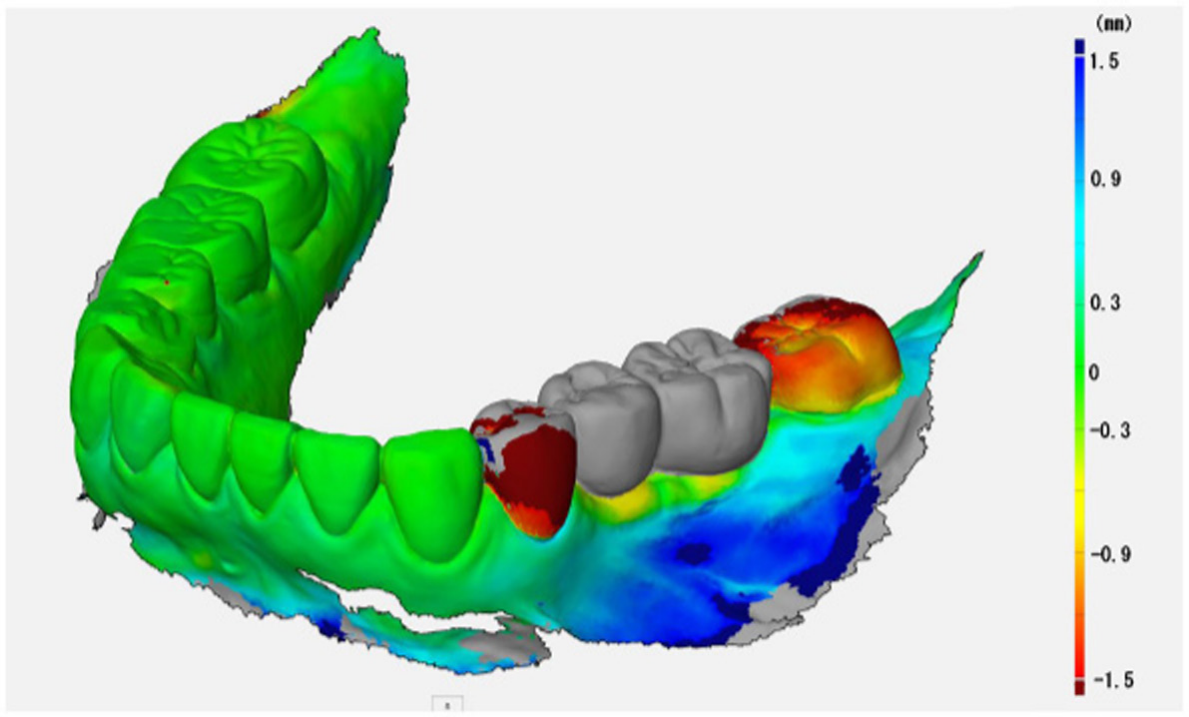
Superimposed intraoral scanning data before implant placement and after prosthetic treatment. Green indicates the same morphology as before implant placement. Red indicates more convex than before implant placement. Blue indicates more concave than before implant placement.

Comparison of soft tissue morphology of intraoral scanning data before implant placement and after prosthodontic treatment showed an increase in peri‐implant tissue after placement of the implant superstructure. This suggests that the peri‐implant tissue stability was due to the prevention of bone resorption around the implants by platform shifting and maintaining the emergence profile by using abutments that did not need to be removed during prosthodontic treatment (Figure 12).^14^

## 4 CONCLUSION

In the present case, using an IOS and CAD/CAM technology as digital technology, implant placement simulation and computer‐guided implant surgery of the edentulous area were performed, and crown prostheses of the remaining teeth and implant superstructures of the edentulous area were simultaneously fabricated to improve impaired masticatory function.

### INFORMED CONSENT

Written informed consent was obtained from the patient to publish this report in accordance with the journal's patient consent policy.

## AUTHOR CONTRIBUTIONS

**Akinori Tasaka:** Project administration. **Hodaka Sasaki:** Supervision. **Atsuro Harada:** Data curation. **Kosei Ito:** Data curation. **Hiro Kobayashi:** Data curation. **Takahiro Shimizu:** Investigation. **Shuichiro Yamashita:** Supervision.

## CONFLICT OF INTEREST STATEMENT

The authors have no conflict of interests to declare.

## Data Availability

None
